# The Hospital’s Need of a Dental Staff

**Published:** 1902-11

**Authors:** A. J. Flanagan

**Affiliations:** Springfield, Mass.


### THE HOSPITAL'S NEED OF A DENTAL STAFF.[1]

BY DR. A. J. FLANAGAN, SPRINGFIELD, MASS.

Mr. President, Members, and Friends of The New York Institute of Stomatology,—About two years ago the essayist was a guest of your society, and presented a paper calling attention to the lack of charitable work by the profession as a whole, and incidentally mentioned that our hospitals throughout the world needed our services also. The editor of the International Dental Journal, some time after this, had an editorial entitled, "Is Dentistry a Charitable Profession?" You will find the essay mentioned and the discussion which followed it in the April, 1900, issue, and the editorial in the May, 1900, issue. The editorial was a very able one of nearly five pages, and, to sum up the whole matter at issue, maintained that it was impossible for dentistry to be a charitable profession along the lines recorded in my paper. The last thought in that editorial I will quote: "While the writer is in full sympathy with the general trend of thought in that discussion, it does seem that with our charitable views we should combine the practical possibilities, and these would appear to be confined to the colleges that are alone capable of bringing together the needy, willing to suffer, and the undergraduate willing and eager to work." Not long since the Valley District Dental Society had a banquet, and in the after-dinner talk the old question of dental charity was brought forth by the writer, as apropos to the remarks of one of our esteemed dental editors, who was one of the guests of the evening. The editor answered my remarks in no uncertain manner that evening. You will find much that he said embodied in an editorial in this month's *Items of Interest*. The editorial is entitled, "Dental Fees. Dental Dignity. Dental Charity." There is but one thought of that editorial I will quote: "It is a fact that dentists do less charity work than they should. There are individual exceptions, of course, but aimed at the entire profession the accusation is true." My thanks are due him for his plain, unvarnished statements; however, my sympathy may be extended later, for he may be put "to battle royal" in defence of his honesty and courage in this matter.

---

[1] Read before The New York Institute of Stomatology, May 6, 1902.

As a continuation of the paper of January, 1900, we will take up some of the needs the hospitals have for the dentist. It was my intention to present a rather elaborate paper for this evening, illustrated by drawings and models. I found, however, that it would be impossible to do justice to what had been planned without three hours of your valuable time. This would have prevented the reading of Dr. Williams's paper and the discussion following, for it has always seemed to me that this society should be complimented on one thing above all others,—the able and thorough analysis which you seem to give all papers. The discussion following a paper is frequently productive of greater good than the paper itself. If the future warrants a continuation of my thoughts, embodied in practical models and drawings, you will find me at your service.

The word hospital can be traced back to the Latin word *hospitalis,* and in the early Latin meant a receiving of strangers or guests in need, without reward or recompense. The present would mean that it is a building in which the sick, injured, or infirm are relieved and treated, either at their own expense or more often by charity in whole or in part. A logical reasoning would deduce the thought that a hospital was not a place for a person at ease; it would be the place for a person suffering with what good Dr. Garretson so well described as a *dis.* If one has not ease, it necessarily follows he must have the opposite,—that is, disease. Therefore, in the broadest and truest sense hospitals should be established for the amelioration or cure of beings suffering with disease. This evening's paper deals with the hospital and its work, from this point of reasoning.

Some one has said that "charity is that priceless gem, affixed by God in the human soul, to measure man's allegiance to the highest and holiest laws of heaven." It is that which brings the entire world to that common centre, relationship. Now, charity, to be effective on the broadest and highest lines, needs organization and system of recognized worth. No one institution has a greater need of these requirements than our present home of benevolence, housed under the roof of a hospital. Time was when our hospitals had a decided limited field of usefulness, but if I may be allowed to prophesy for their future, the claim will be made that all specialties of the healing art will not only have to be recognized there, but will have to be part of their working force. The evolution of the world in thought, science, and art has brought forth the specialist; the past has been a field for the man able to do many things "fairly well;" the future is to produce the man able "to do something" better than others. Might we not claim that the past has been the "constructive," and the future will be the "perfective" age. Admitting then the future as the "perfective" age, we will surely see all specialties of the healing art members of the hospital staffs. If dentistry is a specialty of the healing art, the future will have us enrolled there for service, on a broad humanitarian plane. Then, what of the dental staff's field of usefulness?

The general surgeon finds that fractures of the lower jaw are the most troublesome to treat, as he is not able to apply splints in the ordinary manner, owing to numerous muscles displacing the fragments. The many muscles controlling the jaw, tongue, larynx, and neck play small part in this displacement. If the fracture be at the median line, there will be little or no displacement, as the muscles of one side have no advantage over those of the other. The muscles most active are the masseter, internal pterygoid, mylohyoid, geniohyoid, and geniohypoglossus. A decided movement of the head, either rotary or otherwise, will have a tendency to displace the fragments, and the simple acts of deglutition and speaking will frequently do the same. In the act of swallowing the larynx is elevated by the contraction of the hyoid muscles, which are attached to the tongue and the styloid process of the lower jaw. Before the contraction can take place the lower jaw must be fixed to the upper by the muscles which fix the mouth, when the act may be accomplished. Time forbids going further into the action of the muscles in the various forms of fracture. Necessarily, the inferior—from its exposed position, shape, and office—is more exposed to fracture than the superior maxillary bone. You will generally find the location of a fracture depends to a great extent on the direction of the force causing the same and the position of the teeth remaining in the jaw. Fracture of the ramus is rare. Fractured necks of the condyle are rather frequent, and, as a rule, serious, for you may have brain complications. Fractures of the alveoli are common. Fractures may be simple, compound, or comminuted. It is rarely that we have the nerves and vessels involved from fracture. The average case in the past has frequently been treated by the use of a four-tailed bandage, or by wiring the parts together. The teeth were not considered as essential to either health, comfort, or good looks.

The imperfect contour of the face was of small importance. If the parts had union it was of little consequence whether the articulation was good, bad, or indifferent. The question of comfort was never considered. After some years experience in the treatment of such cases, I am firmly convinced that the case is indeed rare which cannot be best served by the employment of the services of an intelligent practitioner of dentistry. The making of correct splints, from correct models, is the only legitimate method to pursue in the vast majority of cases. It is not the province of this paper to describe the manner of making the various splints required; indeed, only fundamentals could be given, for every case has a different application of the fundamentals.

There are many hospitals which desire and employ the services of the dentist in fracture cases, the filling, cleaning, and the extraction of teeth, but when a hint is made of further use, the question, to say the least, is debatable in certain quarters. My paper of January, 1900, called attention to the needs of ordinary dental service in hospitals and institutions of varied nature, and this evening I propose to pass over the line which is not debatable into that which may be.

The medical journals of the last few years have had many articles on the treatment of cleft palate by surgical operations. There seems to be a revival of this sort of surgery. History has proved in the past that many cases of recorded success—at the time of operation—have not been so in later years. In the vast majority of hospital cases the use of an obturator is not considered. Here lies a field of usefulness second to none. To my mind there should be a thorough understanding of each case, from the stand-point of both the operative surgeon and what might be well termed the appliance surgeon. In these newer methods of surgical operation, is history to repeat itself?

Dentistry has several *dernier ressort* causes for so-called "known and obscure" dental troubles. I will mention but two,—"ulceration" and "you have caught cold." Medicine has our congratulations with several, and of which we need mention but two,—"dyspepsia" and "neuralgia." No patient in relating his troubles stands "the ghost of a show" in the argument when these pseudo troubles are hurled at him. It would be safe to say that at least seventy per cent. of so-called "facial neuralgia" has a dental origin. Patients are prone to seek the advice of medical practitioners for these ailments, and in the vast majority of cases no examination is made, and a drug of some kind is prescribed as the only remedy. The average treatment of dyspepsia is equally as bad. No calling can be considered either scientific or ethical which continues to treat symptoms when causes can be found. Is it not honest to say that many cases of dyspepsia have a dental origin? The treatment of symptoms rather than causes has been the means of sending many a poor human being through after-life a drug degenerate.

Institutions caring for the sick have many forms of disease located in the hard and soft tissues of the mouth and adjacent parts. Of these we may well mention diseases of the mucous membrane, dental pulp, and pericementum. There are many varieties and causes, and here the educated and advanced dentist can be consulted with practical results.

The microscopical examination of blood and urine is playing an important *rôle* to-day in detecting the causes of many disorders. I wish to prophesy that the microscopical examination of saliva is going to play an equally important part. The work of Drs. Michaels, of Paris, and Kirk, of Philadelphia, along these lines is sure to be productive of good results. On Friday last I spent a most enjoyable and profitable hour at the University of Pennsylvania viewing some results of saliva study brought forth by Professor Kirk. If the saliva is to be the means of helping general pathology and therapeutics, who is better able for its study and experimentation than the advanced dentist?

Harvard Dental College for some years has paid special attention to the mechanical treatment of fractured jaws and cleft palates under the very able teaching of Dr. P. W. Moriarty. No student of his ever left the college without being thoroughly drilled in the fundamentals of treatment for such cases, and I know of no one man who is deserving of greater recognition in his specialty. If you are interested in fractured jaws and cleft palates I would suggest your obtaining his writings on the subjects. Individually, I owe to Dr. Moriarty a great debt in first interesting me along the lines of hospital interest and work.

A training of the mind is said to be the meaning of education. A training of the mind in the best sense produces him who observes, compares, reflects, and records. At no time in the history of dental science has there been a greater need of the trained mind. We have floating in hazy atmosphere the outlines of many scientific problems, and we need but the master mind to grasp forth and convert them into living actual truths. What is accepted to-day as the truth in knowledge and practice may not be so to-morrow, and after all, was not this the wisdom of the Infinite in very completeness. If to-day sees the completeness of knowledge and environment, who wishes to hail the to-morrow.

